# Correction: To prescribe or not to prescribe? A factorial survey to explore veterinarians’ decision making when prescribing antimicrobials to sheep and beef farmers in the UK

**DOI:** 10.1371/journal.pone.0217156

**Published:** 2019-07-02

**Authors:** The PLOS ONE Staff

## Notice of Republication

This article was republished on May 7, 2019 to correct for errors to the References and reference citations introduced during the typesetting process. The publisher apologizes for the errors. Please download this article again to view the correct version. The originally published, uncorrected article and the republished, corrected article are provided here for reference.

## Supporting information

S1 File Originally published, uncorrected article. (PDF)

S2 File Republished, corrected article. (PDF)
