# Supplementary material for: To prescribe or not to prescribe? A factorial survey to explore veterinarians’ decision making when prescribing antimicrobials to sheep and beef farmers in the UK
Source: PLoS One. 2019 Apr 9;14(4):e0213855. doi: 10.1371/journal.pone.0213855 (PMC6456164; doi:10.1371/journal.pone.0213855)
Supplement: S1 File — (DOCX) [file pone.0213855.s001.docx]

**VET DECISION MAKING SURVEY: ANTIMICROBIAL PRESCRIBING TO SHEEP & BEEF PRODUCERS**

**Section A: About you and your vet practice**

1. **Please indicate your gender.**

Male  Female  Prefer not to say  Other

1. **What is your age group?**

20-30  31-40  41-50  51-60  61-70  71+

1. **What year did you graduate with your veterinary degree?**

Pre 1970  1971-1980  1981-1990  1991-2000 2001-2010  After 2010

1. **From which university did you graduate with your veterinary degree?**

Bristol  Cambridge  Edinburgh  Glasgow  Liverpool  Nottingham  RVC

Other (Please specify) _______________________

1. **How many vets are currently employed in your practice?** (Please provide a number of all vets that are employed at your primary site of work, including all species)

Full time: ________ Part time: ________

1. **What region is your practice in?**

SW England  SE England  Central England  NW England  NE England

N Scotland & Highlands  S & Central Scotland  N Wales  S Wales  N Ireland

1. **What is your position within the practice?**

Practice partner  Associate/Clinical lead  Assistant  Locum

1. **What type of practice are you employed in?**

Farm  Farm & Equine  Farm & Small Animal  Farm, Equine & SA

1. **What type of work are you involved in within your practice?**

Farm  Farm & Equine  Farm & Small Animal  Farm, Equine & SA

1. **What percentage of your time do you spend working with cattle and sheep?**

Dairy Cattle: _______% Beef Cattle: _______% Sheep: _______%

1. **What percentage of your work with sheep and cattle clients is spent in a disease prevention advisory role?**

Dairy Cattle: _______% Beef Cattle & Sheep: _______%

**Section B: More about you**

Below are a list of statements relating to personality traits that may or may not apply to you. In the table below please tick a box to explain how well the pairs of words describe you, even if one characteristic applies more strongly than the other. Answer as honestly as you can. There are no right or wrong answers. Please only **tick** **one box per row**.

***I see myself as:***

| Characteristics | Strongly disagree | Moderately disagree | Disagree a little | Neither agree nor disagree | Agree a little | Agree moderately | Strongly agree |
| --- | --- | --- | --- | --- | --- | --- | --- |
| Extraverted  Enthusiastic |  |  |  |  |  |  |  |
| Critical  Quarrelsome |  |  |  |  |  |  |  |
| Dependable  Self-disciplined |  |  |  |  |  |  |  |
| Anxious  Easily upset |  |  |  |  |  |  |  |
| Open to new experiences  Complex |  |  |  |  |  |  |  |
| Reserved  Quiet |  |  |  |  |  |  |  |
| Sympathetic  Warm |  |  |  |  |  |  |  |
| Disorganised  Careless |  |  |  |  |  |  |  |
| Calm  Emotionally stable |  |  |  |  |  |  |  |
| Conventional  Uncreative |  |  |  |  |  |  |  |

**Section C: Scenarios**

Section C starts over the page. You will be presented with eight different scenarios that may be seen in practice. Changes in each scenario are indicated by ***bold, italic font***. You will be asked to rate the likelihood that the vet in the scenario would prescribe antibiotics, and what percentage of vets you think would prescribe antibiotics.

In all instances, the antibiotic is **licensed for use in that animal**, and is **not a high priority critically important antibiotic (HP-CIA)**.

In the UK, fluoroquinolones, 3rd and 4th generation cephalosporins and colistin are recognised as high priority CIAs, as designated by the European Medicines Agency.

**Scenario 1**

A farmer comes into the vet practice asking for a bottle of particular antibiotic as he ***wants to prevent watery mouth in a group of home-born lambs*** on the farm. The farmer says that they ***use this antibiotic the same time every year*.**

The farmer has been a client at the vet practice for ***10 years and the vet rarely visits the farm. No other vet in the practice has prescribed*** the farmer this antibiotic without a farm visit before, but the farmer ***is happy to pay for a vet visit if needed.***

The vet ***is not running late*** for their afternoon consults, and ***is not confident*** in the farmers’ judgement of disease.

1. **Based only on the information provided above, how likely or unlikely is the vet to prescribe the farmer with the antibiotic** **without visiting the farm first?** (Please circle)

| Definitely would not prescribe | | |  |  | Not sure |  |  | Definitely would prescribe | | |
| --- | --- | --- | --- | --- | --- | --- | --- | --- | --- | --- |
| -5 | **-4** | **-3** | **-2** | **-1** | **0** | **1** | **2** | **3** | **4** | **5** |

1. **What proportion of vets do you think would prescribe this farmer antibiotics** **without visiting the farm first?** (Please indicate by drawing a line in the scale)


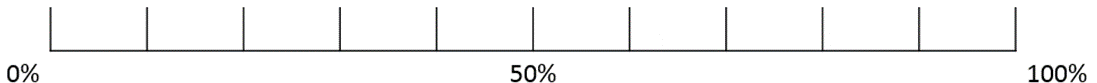


**Scenario 2**

A farmer comes into the vet practice asking for a bottle of particular antibiotic as he ***suspects multiple cases of watery mouth*** on the farm. The farmer says that they ***use this antibiotic the same time every year*.**

The farmer has been a client at the vet practice for ***less than a year and the vet has visited the farm once****.* ***Other vets in the practice have prescribed*** the farmer this antibiotic without a farm visit before, but the farmer ***is happy to pay for a vet visit if needed.***

The vet ***is not running late*** for their afternoon consults, and ***is confident*** in the farmers’ judgement of disease.

1. **Based only on the information provided above, how likely or unlikely is the vet to prescribe the farmer with the antibiotic** **without visiting the farm first?** (Please circle)

| Definitely would not prescribe | | |  |  | Not sure |  |  | Definitely would prescribe | | |
| --- | --- | --- | --- | --- | --- | --- | --- | --- | --- | --- |
| -5 | **-4** | **-3** | **-2** | **-1** | **0** | **1** | **2** | **3** | **4** | **5** |

1. **What proportion of vets do you think would prescribe this farmer antibiotics** **without visiting the farm first?** (Please indicate by drawing a line in the scale)


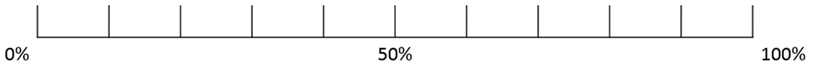


**Scenario 3**

A farmer comes into the vet practice asking for a bottle of particular antibiotic as he ***wants to prevent calf pneumonia in a group of home-born calves*** on the farm. The farmer says that they ***use this antibiotic the same time every year*.**

The farmer has been a client at the vet practice for ***less than a year and the vet has visited the farm once. Other vets in the practice have prescribed*** the farmer this antibiotic without a farm visit before, but the farmer ***is happy to pay for a vet visit if needed.***

The vet ***is running late*** for their afternoon consults, and ***is confident*** in the farmers’ judgement of disease.

1. **Based only on the information provided above, how likely or unlikely is the vet to prescribe the farmer with the antibiotic** **without visiting the farm first?** (Please circle)

| Definitely would not prescribe | | |  |  | Not sure |  |  | Definitely would prescribe | | |
| --- | --- | --- | --- | --- | --- | --- | --- | --- | --- | --- |
| -5 | **-4** | **-3** | **-2** | **-1** | **0** | **1** | **2** | **3** | **4** | **5** |

1. **What proportion of vets do you think would prescribe this farmer antibiotics** **without visiting the farm first?** (Please indicate by drawing a line in the scale)


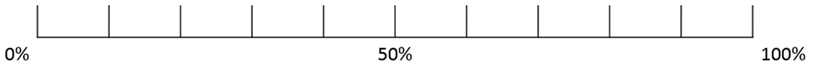


**Scenario 4**

A farmer comes into the vet practice asking for a bottle of particular antibiotic as he ***wants to prevent calf pneumonia in a group of home-born calves*** on the farm. The farmer says that they ***have never used this antibiotic for this reason before, but have used it on the farm for a different reason.***

The farmer has been a client at the vet practice for ***less than a year and the vet has visited the farm once. No other vet in the practice has prescribed*** the farmer this antibiotic without a farm visit before, but the farmer ***does not want to pay for a vet visit.***

The vet ***is running late*** for their afternoon consults, and ***is not confident*** in the farmers’ judgement of disease.

1. **Based only on the information provided above, how likely or unlikely is the vet to prescribe the farmer with the antibiotic** **without visiting the farm first?** (Please circle)

| Definitely would not prescribe | | |  |  | Not sure |  |  | Definitely would prescribe | | |
| --- | --- | --- | --- | --- | --- | --- | --- | --- | --- | --- |
| -5 | **-4** | **-3** | **-2** | **-1** | **0** | **1** | **2** | **3** | **4** | **5** |

1. **What proportion of vets do you think would prescribe this farmer antibiotics** **without visiting the farm first?** (Please indicate by drawing a line in the scale)


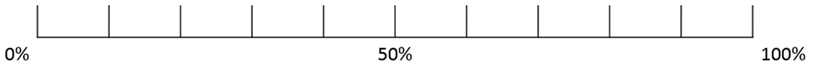


**Scenario 5**

A farmer comes into the vet practice asking for a bottle of particular antibiotic as he ***wants to prevent watery mouth in a group of home-born lambs*** on the farm. The farmer says that they ***have never used this antibiotic for this reason before, but have used it on the farm for a different reason*.**

The farmer has been a client at the vet practice for ***10 years and the vet regularly visits his dairy herd but not as involved with the sheep or beef cattle. Other vets in the practice have prescribed*** the farmer this antibiotic without a farm visit before, but the farmer ***does not want to pay for a vet visit.***

The vet ***is running late*** for their afternoon consults, and ***is confident*** in the farmers’ judgement of disease.

1. **Based only on the information provided above, how likely or unlikely is the vet to prescribe the farmer with the antibiotic** **without visiting the farm first?** (Please circle)

| Definitely would not prescribe | | |  |  | Not sure |  |  | Definitely would prescribe | | |
| --- | --- | --- | --- | --- | --- | --- | --- | --- | --- | --- |
| -5 | **-4** | **-3** | **-2** | **-1** | **0** | **1** | **2** | **3** | **4** | **5** |

1. **What proportion of vets do you think would prescribe this farmer antibiotics** **without visiting the farm first?** (Please indicate by drawing a line in the scale)


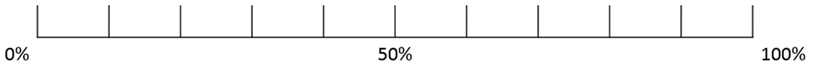


**Scenario 6**

A farmer comes into the vet practice asking for a bottle of particular antibiotic as he ***suspects multiple cases of calf pneumonia*** on the farm. The farmer says that they ***have never used this antibiotic for this reason before, but have used it on the farm for a different reason.***

The farmer has been a client at the vet practice for ***10 years and the vet rarely visits the farm. No other vet in the practice has prescribed*** the farmer this antibiotic without a farm visit before, but the farmer ***does not want to pay for a vet visit.***

The vet ***is not running late*** for their afternoon consults, and ***is confident*** in the farmers’ judgement of disease.

1. **Based only on the information provided above, how likely or unlikely is the vet to prescribe the farmer with the antibiotic** **without visiting the farm first?** (Please circle)

| Definitely would not prescribe | | |  |  | Not sure |  |  | Definitely would prescribe | | |
| --- | --- | --- | --- | --- | --- | --- | --- | --- | --- | --- |
| -5 | **-4** | **-3** | **-2** | **-1** | **0** | **1** | **2** | **3** | **4** | **5** |

1. **What proportion of vets do you think would prescribe this farmer antibiotics** **without visiting the farm first?** (Please indicate by drawing a line in the scale)


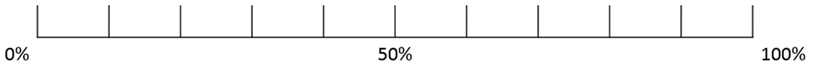


**Scenario 7**

A farmer comes into the vet practice asking for a bottle of particular antibiotic as he ***suspects multiple cases of calf pneumonia*** on the farm. The farmer says that they ***use this antibiotic the same time every year*.**

The farmer has been a client at the vet practice for ***10 years and the vet rarely visits the farm. No other vet in the practice has prescribed*** the farmer this antibiotic without a farm visit before, but the farmer ***is happy to pay for a vet visit if needed.***

The vet ***is not running late*** for their afternoon consults, and ***is not confident*** in the farmers’ judgement of disease.

1. **Based only on the information provided above, how likely or unlikely is the vet to prescribe the farmer with the antibiotic** **without visiting the farm first?** (Please circle)

| Definitely would not prescribe | | |  |  | Not sure |  |  | Definitely would prescribe | | |
| --- | --- | --- | --- | --- | --- | --- | --- | --- | --- | --- |
| -5 | **-4** | **-3** | **-2** | **-1** | **0** | **1** | **2** | **3** | **4** | **5** |

1. **What proportion of vets do you think would prescribe this farmer antibiotics** **without visiting the farm first?** (Please indicate by drawing a line in the scale)


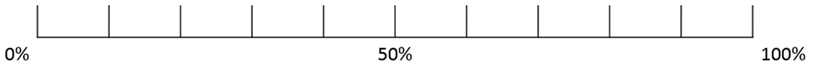


**Scenario 8**

A farmer comes into the vet practice asking for a bottle of particular antibiotic as he ***suspects multiple cases of watery mouth*** on the farm. The farmer says that they ***have never used this antibiotic for this reason before, but have used it on the farm for a different reason*.**

The farmer has been a client at the vet practice for ***10 years and the vet regularly visits his dairy herd but not as involved with the sheep or beef cattle. No other vet in the practice has prescribed*** the farmer this antibiotic without a farm visit before, but the farmer ***does not want to pay for a vet visit****.*

The vet ***is running late*** for their afternoon consults, and ***is not confident*** in the farmers’ judgement of disease.

1. **Based only on the information provided above, how likely or unlikely is the vet to prescribe the farmer with the antibiotic** **without visiting the farm first?** (Please circle)

| Definitely would not prescribe | | |  |  | Not sure |  |  | Definitely would prescribe | | |
| --- | --- | --- | --- | --- | --- | --- | --- | --- | --- | --- |
| -5 | **-4** | **-3** | **-2** | **-1** | **0** | **1** | **2** | **3** | **4** | **5** |

1. **What proportion of vets do you think would prescribe this farmer antibiotics** **without visiting the farm first?** (Please indicate by drawing a line in the scale)


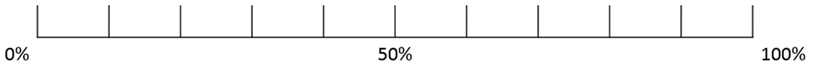


**Section D: Your opinions on antibiotics**

In the previous scenarios, the antibiotic requested was licensed in cattle and sheep, and not a high priority critically important antibiotic (HP-CIA).

In the UK, fluoroquinolones, 3rd and 4th generation cephalosporins and colistin are recognised as high priority CIAs, as designated by the European Medicines Agency.

Imagine if you had been requested to prescribe the antibiotics in the table below. Please indicate, using the tick boxes provided, whether your decision would have been any different.

| Requested antibiotic | Much less likely to prescribe | Moderately less likely to prescribe | A little less likely to prescribe | Remain the same | A little more likely to prescribe | Moderately more likely to prescribe | Much more likely to prescribe |
| --- | --- | --- | --- | --- | --- | --- | --- |
| An antibiotic that was not licensed for that species |  |  |  |  |  |  |  |
| A high priority critically important antibiotic (HP-CIA) |  |  |  |  |  |  |  |
| A bottle of marbofloxacin for calf pneumonia |  |  |  |  |  |  |  |
| An enrofloxacin oral doser for watery mouth |  |  |  |  |  |  |  |
| Oxytetracycline tablets for watery mouth |  |  |  |  |  |  |  |

**Section E: Further research**

1. **Would you be willing to participate in further research into antimicrobial use for the University of Nottingham?** (e.g. sharing veterinary sales records)

Yes  No

1. **If you said yes, please provide the phone number and/or email address you would prefer to be contacted on:**

Phone number: _______________________

Email address: ____________________________

**Thank you for participating in this survey.**
