# Supplementary material for: Correction: To prescribe or not to prescribe? A factorial survey to explore veterinarians’ decision making when prescribing antimicrobials to sheep and beef farmers in the UK
Source: PLoS One. 2019 Jul 2;14(7):e0217156. doi: 10.1371/journal.pone.0217156 (PMC6605644; doi:10.1371/journal.pone.0217156)
Supplement: S2 File — (PDF) [file pone.0217156.s002.pdf]

PLOS ONE

RESEARCH ARTICLE

To prescribe or not to prescribe? A factorial survey to explore veterinarians' decision making when prescribing antimicrobials to sheep and beef farmers in the UK

Charlotte Doidge*, Chris Hudson, Fiona Lovatt, Jasmeet Kaler

School of Veterinary Medicine and Science, University of Nottingham, Leicestershire, England

* charlotte.doidge@nottingham.ac.uk

Check for updates

OPEN ACCESS

Citation: Doidge C, Hudson C, Lovatt F, Kaler J (2019) To prescribe or not to prescribe? A factorial survey to explore veterinarians' decision making when prescribing antimicrobials to sheep and beef farmers in the UK. PLoS ONE 14(4): e0213855. https://doi.org/10.1371/journal.pone.0213855

Editor: Simon Russell Clegg, University of Lincoln, UNITED KINGDOM

Received: January 17, 2019

Accepted: March 3, 2019

Published: April 9, 2019

Copyright: © 2019 Doidge et al. This is an open access article distributed under the terms of the Creative Commons Attribution License, which permits unrestricted use, distribution, and reproduction in any medium, provided the original author and source are credited.

Data Availability Statement: Data is available under the CC0 license at https://doi.org/10.7910/DVN/TRR6JJ.

Funding: CD was supported by Agricultural and Horticultural Development Board Beef and Lamb PhD studentship (Ref 61110063) URL https://ahdb.org.uk/beef-lamb. The funders had no role in study design, data collection and analysis, decision to publish, or preparation of the manuscript.

Competing interests: The authors have declared that no competing interests exist.

Abstract

Resistance to antimicrobials is one of the biggest challenges worldwide for public health. A key strategy for tackling this is ensuring judicious use of antimicrobials in human and veterinary medicine. Whilst there are many studies in human medicine investigating prescribing behaviour of doctors, there is limited work to understand what factors influence veterinarian prescribing behaviour. Veterinarians often prescribe antimicrobials to sheep and beef farmers in contexts other than at a clinical consultation, and decision-making behind this has not been explored. The aim of this study was to measure, for the first time, the influence of factors from social theories on veterinarians' decision to prescribe antimicrobials to sheep and beef farmers without a clinical consultation, using a factorial survey approach. Respondents were presented with eight vignette scenarios, where a farmer asks for antimicrobials at the veterinary practice. Seven factors, identified from constructs of social theories, were included in the vignettes. Random intercept and random slope models were built to estimate the effects of the vignette factors and vet characteristics on the respondents' willingness to prescribe ratings. A total of 306 surveys were completed. The vignette factors: case type, farmer relationship, other veterinarians in practice, time pressure, habit, willingness to pay, and confidence in the farmer, were significant in the decision to prescribe. Confidence in the farmer was the most influential vignette variable, and was included as a random slope effect. Respondent variables with significant influence on the decision to prescribe were agreeableness personality score, region of veterinary practice, and presence of a small animal department. These influential factors could be considered to target interventions in beef and sheep farm animal veterinary practice for improved antimicrobial stewardship.

Introduction

The emergence of multi-drug resistant bacteria has developed into a world-wide public health concern. Whilst research so far has been unable to quantify the risk of the transfer of antimicrobial resistant bacteria from animals to humans, there is evidence that a reduction in antimicrobial use can lead to a reduction in antimicrobial resistance in animals [1, 2]. A "One

Health" perspective is required to tackle the rising threat of antimicrobial resistance, which involves humans, animals, and the environment. Therefore, one of the priorities is to ensure responsible use of antimicrobials in agriculture, which can be achieved through understanding the decision-making process around prescribing antimicrobials [3].

Studies have shown that veterinarians were the most influential source of information for farmers' antimicrobial use (AMU) [4]. In the UK, veterinary antibiotics are a prescription only medicine (POM-V) which can only be prescribed by a veterinarian. Veterinarians generally have very little presence on sheep farms in particular, and are often only called onto the farm in emergencies [5]. There is a lack of information on the use of veterinarians by UK beef farmers, but it may be a similar situation to sheep, due to the overlap in enterprises. For example, a third of less favoured area livestock farmers–which are most often sheep and cattle rearing–did not consult their veterinarian for a year or more [6]. According to the Code of Professional Conduct for Veterinary Surgeons, veterinarians can prescribe as long as the animal has been seen "recently enough or often enough for the veterinary surgeon to have personal knowledge of the condition of the animal or current health status of the herd or flock to make a diagnosis". Therefore, it is not uncommon for farmers to receive antibiotics from veterinarians without a clinical consultation in which animals are examined at the time of prescription.

The sheep and beef sectors in the UK are highly interlinked, with 69% of sheep farmers owning a beef enterprise [7]. This makes it difficult to distinguish use between the species when multi-species antimicrobials are used. A study from a sample of beef-only farms suggests an average antimicrobial usage of 19mg/kg, which is lower than the 37mg/kg average across all food-producing animals [8]. Despite this, a previous study using veterinary prescription records estimated that there is huge variability in usage among sheep farms [9], which may indicate that some farms may be using antimicrobials unnecessarily, and veterinarians prescribing inappropriately. The study also showed that 21% of variation in the usage could be attributed to the veterinary practice. Coupled with antibiotics having POM-V status, this suggests that veterinarian prescription behaviour plays an important role in AMU at the farm level.

In order to investigate what is causing these differences in veterinary prescription, studies into the human behaviour regarding AMU are necessary. There has been limited research in the area of veterinarian decision-making. Most of the work on this topic used qualitative methods, [10–13] to explore the common factors that influence prescribing behaviour. The studies have identified psychosocial factors such as relationship with clients, previous experience, and risk avoidance. While these studies give some insights into what factors influence veterinary behaviour towards prescribing antimicrobials, it is unknown how these factors are represented in a larger population of veterinarians, and the relative importance of these factors in influencing veterinarian's decisions. The limited number of quantitative studies that explore veterinarian antimicrobial prescribing behaviours over a larger population often focus on enterprises other than sheep and beef, such as pigs [14, 15]. The few studies that do include prescribing to beef farmers may over-simplify the prescribing decision through asking about each potential factor individually [16–18]. Therefore, the ecological validity is compromised, as the decision does not mimic "real-life" conditions. As of yet, veterinary antimicrobial prescribing behaviour towards sheep farmers in the UK has not been investigated. In addition, since the work exploring veterinarian prescribing behaviour has not used any underpinning theoretical framework, the role of additional factors is unknown. Finally, due to the lack of veterinarian involvement on sheep and potentially beef farms, prescribing in contexts other than clinical consultation is common [6]; veterinarians prescribing behaviour in this setting has not been explored before.

Physician prescribing behaviour has been much more comprehensively researched when compared to veterinarian prescribing behaviour, and has identified the influence of factors such as other staff members, pressure from patients, and past behaviour [19, 20]. This work

relied on a range of social-cognitive theories to study healthcare professional's behaviours, with the Theory of Planned Behaviour (TPB) most commonly used [21]. There is evidence from the health psychology domain to suggest that cross-theory constructs could explain more variation in behaviour than using constructs from a single theory; this has not been applied or tested for veterinarian decision-making [22]. One challenge in understanding decision-making is that decisions are complex and are not made in a social vacuum; resulting in social desirability bias. This is of particular importance for measuring antimicrobial prescription behaviour, due to the public and media attention AMU in agriculture receives. To address this, factorial survey methods have been used widely in social care [23], criminology [24], and clinical decision-making, however, are yet to be used veterinary decision-making [25]. The aim of this study was to use, for the first time in veterinary decision-making, a factorial survey approach to measure the influence of factors from various social theories on a veterinarian's decision to prescribe antimicrobials to sheep and beef farmers, without a clinical consultation first.

Methods

Survey design

This study used a factorial survey approach to measure veterinarians prescribing decisions. An overview of this approach is provided by Taylor [26]. Respondents were presented with a series of eight vignettes which mimic a real life scenario. Each vignette was comprised of seven factors, where the factors had between two and four possible levels. In each vignette, the order of the factors remained the same, but the levels were experimentally varied. A total of 384 unique vignette combinations were possible. The vignette combinations were randomly selected using a resolution V D-efficient design from a sample of the original 384 [27].

Scenario and dependent variables. The scenario used in the vignettes was presented as a farmer coming into the veterinary practice and requesting some antibiotics from the veterinarian. Scenarios were reviewed by authors who are trained veterinarians to check for plausibility. The respondents were informed that in all scenarios the farmer is requesting an antibiotic that is licensed for use in sheep and cattle, and is not a high priority critically important antibiotic (CIA). To reduce ambiguity, the European Medicines Agency definition of CIAs was included, in which fluoroquinolones, 3rd and 4th generation cephalosporins, and colistin were recognised as high priority CIAs.

The respondents were asked to rate two questions relating to this scenario at the end of each vignette (Fig 1):

1. (Outcome A) how likely the veterinarian in the scenario would prescribe antibiotics to the farmer, from -5 (definitely would not prescribe) to +5 (definitely would prescribe);
2. (Outcome B) the percentage of veterinarians that they think would prescribe in that situation, from 0% to 100%.

The rating scale -5 to +5 was used as with previous factorial survey research [28]. Outcome B was asked to allow for the identification of the perceived prescribing norm.

Vignette predictor variables. Variables included in the vignettes represented key constructs from multiple social theories, presented below in Table 1. These variables were identified from previous research in veterinarian and physician decision-making [13, 18, 21]. Watery mouth in lambs and calf pneumonia were chosen as diseases for the "knowledge of case" variables as they are common neonatal diseases where antibiotics may be given either for treatment or prevention [29, 30].

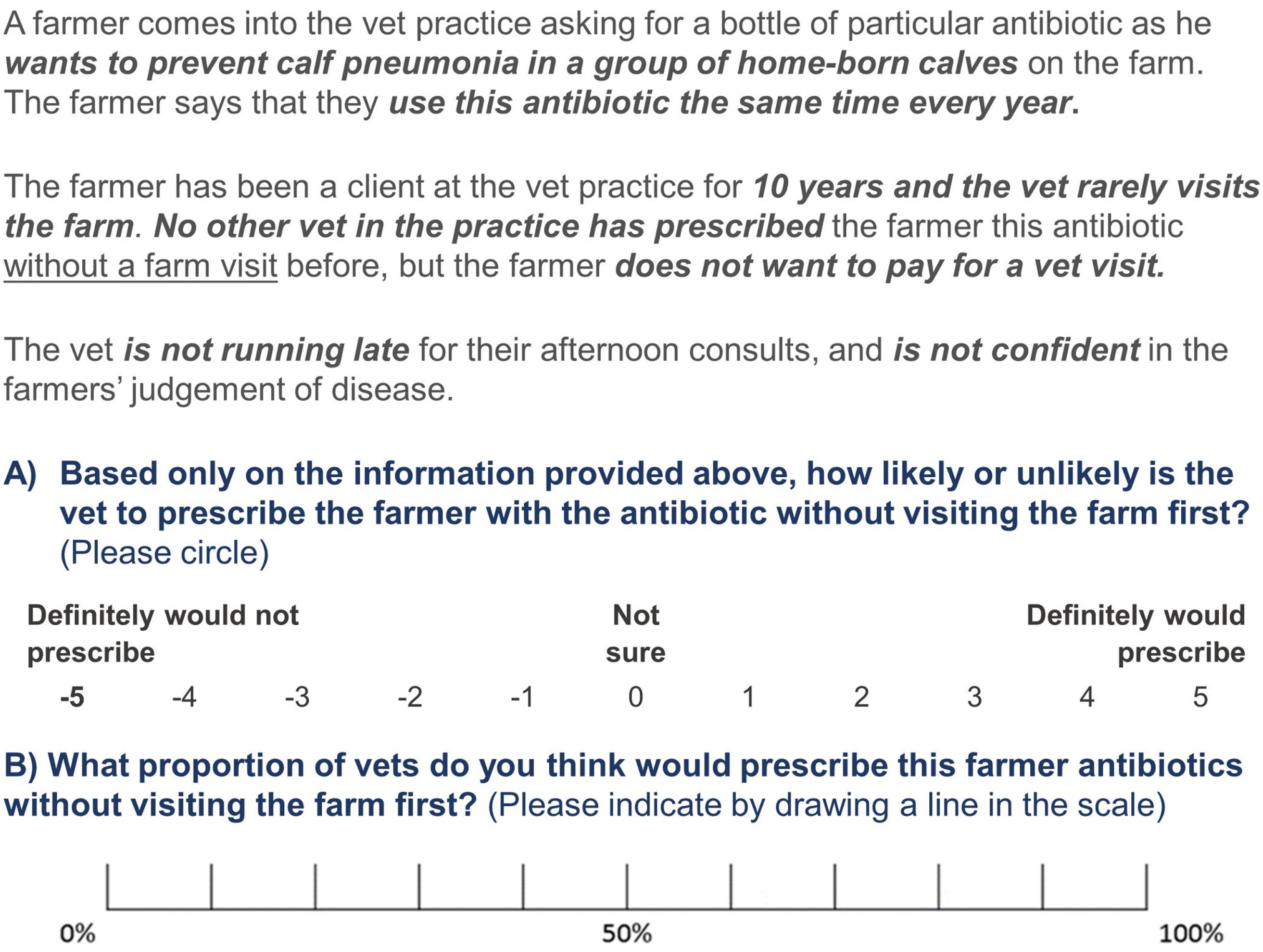

A farmer comes into the vet practice asking for a bottle of particular antibiotic as he ***wants to prevent calf pneumonia in a group of home-born calves*** on the farm. The farmer says that they ***use this antibiotic the same time every year.***

The farmer has been a client at the vet practice for ***10 years and the vet rarely visits the farm***. ***No other vet in the practice has prescribed*** the farmer this antibiotic without a farm visit before, but the farmer ***does not want to pay for a vet visit.***

The vet ***is not running late*** for their afternoon consults, and ***is not confident*** in the farmers' judgement of disease.

A) Based only on the information provided above, how likely or unlikely is the vet to prescribe the farmer with the antibiotic without visiting the farm first? (Please circle)

Definitely would not prescribe					Not sure					Definitely would prescribe
-5	-4	-3	-2	-1	0	1	2	3	4	5

B) What proportion of vets do you think would prescribe this farmer antibiotics without visiting the farm first? (Please indicate by drawing a line in the scale)

0% 50% 100%

Fig 1. Example vignette used in the factorial survey.

https://doi.org/10.1371/journal.pone.0213855.g001

Other predictor variables. As well as the vignette variables, closed questions on respondent characteristics were included in the survey: age, gender, university of graduation, year of graduation, position in practice, and time spent with sheep and cattle. Respondents were also asked questions about their practice: region, practice type and number of veterinarians employed.

The ten point personality inventory was used to obtain personality scores for each veterinarian for extraversion, agreeableness, conscientiousness, emotional stability, and openness to experiences [36]. Scores of corresponding statements were averaged to produce an overall score for "The Big Five" personality traits using methods stated by Gosling, Rentfrow and Swann [36].

Study sample. The anonymous survey took approximately fifteen minutes to complete and was piloted on eight farm veterinarians, all of which worked in practice, three of which also worked in academia. After changes were made, all 813 UK veterinary practices who treat sheep and cattle according to the RCVS website were mailed the English-language paper-based survey in March 2018.

The estimated sample size was calculated using G*Power for a multiple regression analysis with up to 32 predictor variables [37]. For a small effect size of 0.02 and power of 0.95 a sample

Table 1. Variables included in the factorial survey vignettes, and the theoretical constructs they represent.

Variable	Level	Theory construct
**Knowledge of case**	1. Farmer suspects case of calf pneumonia.<br>2. Farmer suspects case of watery mouth.<br>3. Farmer wants to prevent watery mouth.<br>4. Farmer wants to prevent calf pneumonia.	Epistemological–dependent on their existing knowledge on antimicrobials
**Farmer relationship influence**	1. The farmer is a long term client and the veterinarian regularly visits his dairy herd but not as involved with the sheep or beef cattle.<br>2. The farmer is a long term client and the veterinarian rarely visits the farm.<br>3. The farmer is a new client.	Commitment in commitment-trust theory [31]
**Veterinarian influence**	1. Other veterinarians in the practice have given the farmer this antibiotic before without consultation.<br>2. No other veterinarian in the practice has given the farmer this antibiotic before without consultation.	Social influence construct found in: TPB [32], Theory of Interpersonal Behaviour [33], Norm Theory [34]
**Time pressure**	1. No time pressure for prescription.<br>2. Running late for afternoon surgery consults.	Can alter decision-making process according to Decision Field Theory [35]
**Habit**	1. The farmer comes in for the same medication the same time every year.<br>2. The farmer has never used the antibiotic for this reason before.	Component of Theory of Interpersonal Behaviour
**Willingness to pay**	1. Farmer does not want to pay for a veterinary visit.<br>2. Farmer says he is happy for a veterinary visit.	Perceived behavioural control in TPB, and facilitating conditions in Theory of Interpersonal Behaviour
**Confidence**	1. The veterinarian is confident in the farmers' judgement of the disease.<br>2. The veterinarian is not confident in the farmers' judgement of disease.	Trust in commitment-trust theory

https://doi.org/10.1371/journal.pone.0213855.t001

of 1842 was required. As the unit of analysis was the vignette, and each respondent rates eight vignettes, a sample of 230 respondents was required for analysis.

The study was approved by School of Veterinary Medicine and Science Ethics Committee (no 1850 160916).

Data analysis

Data handling and univariable analysis. All data were processed and checked for errors using Stata 15.1 software (Stata SE/15.1, Stata Corp., College Station, TX, USA). Descriptive statistics and univariable analysis were carried out to explore the data. Regions of veterinary practice, personality score and age were used as categorical variables. For the likelihood to prescribe (outcome A), the original outcome rating scale of -5 to +5 was recoded to a 1–11 scale. For percentage of veterinarians that they thought would prescribe (outcome B), the respondents rating of 0–100% were grouped to the nearest 10% and coded from 1–10. Both outcomes were treated as continuous variables as recommended by Auspurg and Hinz [28].

Multilevel modelling. For each outcome, multilevel linear models were built using MLwiN 3.02 [38]. In all models a stepwise model building approach was used, where only variables with $p \leq 0.05$ were selected to remain in the models. First random intercept models (Model 1A and Model 1B) were built and took the form of Eq 1;

$$y_{ij} = \beta_{0ij} + \beta_{x1} vignettevariables_{ij} + \beta_{z1} vetvariables_{j} + u_{0j} + e_{0ij} \quad \text{(Eq1)}$$

Then, a random slope term was included for the confidence vignette variable (Model 2A and Model 2B), to allow for between-respondent variation for the influence of confidence on vignette ratings. Therefore, the random slope models took the form of Eq 2;

$$y_{ij} = \beta_{0ij} + \beta_{x1} vignettevariables_{ij} + \beta_{z1} vetvariables_{j} + u_{0j} + u_{1j} confidence_{ij} + e_{0ij} \quad \text{(Eq2)}$$

Where y_{ij} was the outcome of likelihood to prescribe rating (Outcome A) or percentage of veterinarians who would prescribe (Outcome B) for the ith vignette situation, rated by the jth

veterinarian; β_{0ij} was the intercept; β_{x1} was the coefficient for the effect of a unit increase of the predictor x_{ij} on the outcome; β_{z1} was the coefficient for the effect of a unit increase of the predictor z_j on the outcome; u_{0j} and e_{0ij} are the random effects at the veterinarian and vignette levels, respectively; and u_{1j} was the random term for the coefficient of the confidence variable.

The fit of the final models was evaluated by inspection of the residuals at each level to check they followed a normal distribution. K-fold cross validation was carried out in Stata 15.1 software to check model performance and validity and ensure consistency of results between folds. The Mean Absolute Error (MAE) was calculated. To compare model performance of the random intercept and random slope models, Akaike Information Criterion (AIC) was calculated and a likelihood ratio test was carried out. Additionally, the Variance Partitioning Coefficient (VPC) was calculated for each model to determine the proportion of variance attributable to the respondent level. The R^2 values were calculated based on methods of Nakagawa and Schielzeth [39] and Johnson [40] using the MuMIn R package in RStudio [41, 42].

Results

Respondent demographics

A total of 306 surveys were completed and were received from 199 different veterinary practices. This gave a practice response rate of 24%. There were a total of 2448 vignette evaluations. The respondent characteristics are described in Table 2. Of the respondents, 47.8% were female. Almost half (48.5%) of the respondents were aged 30 or under, with over half (52.8%) graduating after 2010. The majority of respondents were Assistants (61.6%), 25.3% were Practice Partners and 11.8% were Associates or Clinical Leads within their practice.

Multilevel models

Model 1A, a random intercept model for the respondents' likelihood to prescribe ratings (Outcome A), and Model 2A, including a random slope effect for the variable of veterinarians confidence in farmer's judgement, are presented in Table 3. Compared to a case of suspected calf pneumonia, veterinarians rated the likelihood to prescribe almost half a point higher for a case of suspected watery mouth (β = 0.442, CI = 0.191, 0.691), and one point lower for prevention of calf pneumonia (β = -0.959, CI = -1.207, -0.710); with ratings for prevention of watery mouth not significantly different to suspected calf pneumonia (β = 0.224, CI = -0.028, 0.475). Variables such as "the veterinarian is not confident in the farmer's judgement of disease" (β = -1.752, CI = -1.931, -1.573), "farmer says he is happy for a veterinary visit" (β = -0.873, CI = -1.049, -0.697), "farmer has never used the antibiotic for this reason before" (β = -0.565, CI = -0.741, -0.388), "farmer has been a client for less than a year" (β = -0.339, CI = -0.553, -0.125), "practice has a small animal department" (β = -0.491, CI = -0.959, -0.002), and "agreeableness score ≥6" (β = -0.624, CI = -1.156, -0.092) had a significant negative impact on the likelihood to prescribe rating. Variables such as "other veterinarians in the practice have prescribed the farmer this antibiotic before without consultation" (β = 0.686, CI = 0.509, 0.863), "farmer is a client of 10 years and the veterinarian regularly visits his dairy herd" (β = 0.612, CI = 0.392, 0.831), and "veterinarian is not running late for afternoon consults" (β = 0.510, CI = 0.334, 0.687) had a significant positive impact on likelihood to prescribe ratings.

Model 1B, a random intercept model for the respondents expected percentage of veterinarians who would prescribe (Outcome B), and Model 2B, including a random slope effect for the variable of veterinarians confidence in farmer's judgement, are presented in Table 4. Models for outcome B included the same variables with the same positive or negative associations as models for outcome A, as described above. However, "small animal practice" was removed from the model as it was no longer significant. Age was dichotomised at 31 years because just

Table 2. Descriptive statistics of respondent characteristics.

Characteristic	Percentage %	Number	
**Gender of respondent**			
Male	51.8	158	
Female	47.8	146	
Other	0.3	1	
**Age of respondent**			
30 or under	48.5	148	
31–40	24.6	75	
41 and over	26.9	82	
**Year of graduation**			
After 2010	52.8	161	
2001–2010	21.3	65	
Before 2001	25.9	79	
**University of graduation**			
Bristol	12.5	38	
Cambridge	5.6	17	
Dublin	4.3	13	
Edinburgh	16.7	51	
Glasgow	15.4	47	
Liverpool	12.8	39	
Nottingham	7.5	23	
RVC	13.1	40	
Other	12.1	37	
**Region of veterinary practice**			
Central England	10.5	32	
North East England	8.6	26	
North West England	16.1	49	
South East England	10.9	33	
South West England	21.7	66	
Northern Scotland & the Highlands	10.9	33	
Southern & Central Scotland	7.2	22	
Wales	7.9	24	
Northern Ireland	6.3	19	
**Position in practice**			
Assistant	61.6	188	
Associate/clinical lead	11.8	36	
Locum	1.3	4	
Practice partner	25.3	77	
**Practice type/Work type**			
Farm	17.7/34.1	54/104	
Farm & equine	6.6/11.8	20/36	
Farm & small animal	14.8/13.1	45/40	
Farm, equine & small animal	61.0/41.0	186/125	
**Number of veterinarians in practice**	**Median**	**25% IQR**	**75% IQR**
Full time	6	4	10
Part time	1	0	3
**% time spent**	**Median**	**25% IQR**	**75% IQR**
With dairy cattle	30	5	60

(*Continued*)

Table 2. (Continued)

Characteristic	Percentage %	Number	
With beef cattle	20	10	33
With sheep	10	5	20
In an advisory role for dairy	20	3	40
In an advisory role for beef & sheep	20	10	40

IQR = interquartile range

https://doi.org/10.1371/journal.pone.0213855.t002

under half of the study population were under the age of 31. This variable for age was added to the models for outcome B as it was found to be significant, where "age ≥31 years" (β = -0.438, CI = -0.800, -0.076) had a significant negative impact on percentage of veterinarians ratings.

For both outcomes, residuals at both levels indicated a normal distribution. Analysis showed that the AIC scores were lower for the random slope models, and the likelihood ratio tests were significant ($p \leq 0.001$), indicating improved model fit. The VPC for the models indicated that 33.5%-36.6% of variability in prescribing decisions was attributed due to differences among vets. R^2 values showed that the models explained 21–24% of variance. The negative covariance between the slope and intercept values indicated that veterinarians who were less likely to prescribe overall were more influenced by the "confidence in farmers' judgement" variable.

From the results obtained from the models to explain the veterinarian's likelihood to prescribe antimicrobials, and the percentage of veterinarian's which respondents expected to prescribe antimicrobials, a theoretical framework was produced using all significant factors (Fig 2).

Discussion

To the authors' knowledge, this is the first and only study to date in veterinary decision-making that investigated (a) the impact of key constructs from multiple social theories on veterinarian decision-making; (b) the prescription decision of antimicrobials to both sheep and beef farmers; and (c) decisions around prescription in contexts other than clinical consultation. The novel aspect of this work is that it identified a range of different psychosocial factors that significantly influence the veterinarian's decision to prescribe antimicrobials to sheep and beef farmers. These factors could be considered as a framework for understanding and influencing veterinarian decision-making regarding antimicrobial prescription to sheep and beef farmers.

The most influential factor on the decision to prescribe was confidence in the farmers' judgement of disease. The results suggest that impact of this variable was different among veterinarians i.e. veterinarians whose vignette ratings were largely influenced by the confidence variable will have a steeper slope. Similarly, Coyne et al [15]identified confidence that farm staff would use antimicrobials responsibly as a positive driver for antimicrobial prescription by farm animal veterinarians. Morgan and Hunt [31] theorised that trust and commitment were the heart of a successful business-client relationship. They highlight that as part of their commitment-trust theory, confidence is integral to the definition of trust. Whilst previous research identified the importance of farmers trust in veterinarians [43, 44], there has been little research into the importance of veterinarians trust in farmers. This is an area which needs further investigation to fully understand how trust develops in this relationship.

In this study, farmer relationship was shown to be influential, as veterinarians were less likely to prescribe to a new client when compared to a long-term client. This factor was

Table 3. Random slope and random intercept models to explain the veterinarian's likelihood to prescribe antimicrobials to a sheep and beef farmer vignette ratings (Outcome A).

	Model 1A		Model 2A	
**Outcome A**	**β**	**SE**	**β**	**SE**
**Case type**				
Farmer suspects calf pneumonia	ref		ref	
Farmer suspects watery mouth	0.442**	0.128	0.401**	0.125
Farmer wants to prevent calf pneumonia	-0.959***	0.127	-0.994***	0.125
Farmer wants to prevent watery mouth	0.224	0.128	0.190	0.126
**Farmer relationship**				
The farmer is a client of 10 years and the veterinarian rarely visits the farm	ref		ref	
The farmer is a client of 10 years and the veterinarian regularly visits his dairy herd	0.612***	0.112	0.614***	0.111
The farmer has been a client for less than a year	-0.339**	0.109	-0.330**	0.107
**Other veterinarians in practice**				
No other veterinarian in the practice has prescribed the farmer this antibiotic before without consultation	ref		ref	
Other veterinarians in the practice have prescribed the farmer this antibiotic before without consultation	0.686***	0.090	0.688***	0.090
**Time pressure**				
The veterinarian is running late for afternoon consults	ref		ref	
The veterinarian is not running late for afternoon consults	0.510***	0.090	0.520***	0.089
**Habit**				
The farmer uses this antibiotic the same time every year	ref		ref	
The farmer has never used the antibiotic for this reason before	-0.565***	0.090	-0.580***	0.088
**Willingness to pay**				
Farmer does not want to pay for a veterinary visit	ref		ref	
Farmer says he is happy for a veterinary visit	-0.873***	0.090	-0.865***	0.088
**Confidence**				
The veterinarian is confident in the farmers' judgement of the disease	ref		ref	
The veterinarian is not confident in the farmers' judgement of disease	-1.752***	0.091	-1.751***	0.112
**Practice type**				
No small animal practice	ref		ref	
Yes small animal practice	-0.491*	0.239	-0.496*	0.239
**Region**				
South East England	ref		ref	
Northern Ireland	3.575***	0.525	3.615***	0.525
Scotland, North West England	1.344***	0.346	1.355***	0.346
Wales, Central, North East, and South West England	0.847*	0.330	0.851*	0.331
**Agreeableness score**				
< = 4	ref		ref	
4.5–5.5	-0.408	0.257	-0.394	0.258
> = 6	-0.624*	0.271	-0.620*	0.272
**Intercept**	6.289	0.412	6.301	0.413
**Covariance (slope, intercept)**			-0.732	0.245
**Log likelihood**	-5205.599		-5189.168	
**AIC**	10449.20		10420.34	
	**Marginal**	**Cond.**	**Marginal**	**Cond.**
**R^2**	0.240	0.495	0.241	0.538
**VPC**	0.335			
**MAE**	1.760		1.626	
**N Vignettes**	2281		2281	

(*Continued*)

Table 3. (Continued)

	Model 1A		Model 2A	
Outcome A	β	SE	β	SE
N Respondents	287		287	

*p≤0.05

**p≤0.01

***p≤0.001. SE = Standard Error; Cond. = Conditional

https://doi.org/10.1371/journal.pone.0213855.t003

included to account for the commitment construct in the commitment-trust theory, where one of the drivers for commitment is the relationship termination costs. The results from this study suggests that vets are committed to maintaining long-term relationship with their clients, and may prescribe to long-term clients at their request. Relationship with the client was a major influence of prescribing for pig veterinarians [10, 13], and cattle veterinarians [18]. However, this influence appeared to be due to perceived client demand, rather than maintenance of client relationship.

Veterinarians were more likely to prescribe when the farmer received regular veterinary visits. This may be because they have more recent knowledge of the condition of the animal or current health status of the herd or flock. In the UK, the farm animal veterinary profession is moving towards a more advisory role, where their work is oriented to managing herd and flock health, rather than treating sick individual animals [45]. In the scenario, regular veterinary visits may influence the decision to prescribe as it may suggest that a one-to-one relationship between the farmer and veterinarian exists. This one-to-one relationship is recommended in the RONAFA opinion towards a reduction in antimicrobial prescribing, through drawing up preventative herd or flock health plans and benchmarking [46].

The respondents were significantly more likely to prescribe, and also expected more veterinarians to do so, when the veterinarian in scenarios was not running late. This is contradictory to previous research in physician prescription behaviour–whereby physicians stated that they are more likely to prescribe antimicrobials when they are under time pressure [47]. A prescription can be used as a method to end the consultation faster [48]. One possible reason for this difference is because veterinarians in the scenario are prescribing without having a clinical consultation. This study suggests that veterinarians may feel more comfortable prescribing antimicrobials to farmers if they have more time to discuss the matter with them. In a previous study, over 50% of cattle veterinarians stated that time pressure would have no effect on their prescribing behaviour [18]. However, the previous survey used direct questioning and so the less direct questioning in the current study possibly uncovered some bias in the previous study.

Respondents were significantly more likely to prescribe when another veterinarian in practice had prescribed to the farmer before. It is well known that in human medicine, doctors' prescribing decisions are heavily influenced by relationships with team members [49]. The results of this study suggest that veterinarians may also succumb to similar prescribing norms. Indeed, it has previously been identified in pig practice that more senior team members of the veterinary practice may pressurise junior members to prescribe antimicrobials to their "good" clients [13].

Veterinarians were significantly more likely to prescribe if the farmer used that antimicrobial the same time every year. The two diseases used in the scenarios, watery mouth in lambs and calf pneumonia, tend to be seasonal, and farmers may stock up on antibiotics around lambing or weaning time in calves. This may suggest that veterinarians are more likely to

Table 4. Random slope and random intercept models to explain the percentage of vets which respondents expected to prescribe antimicrobials in the vignette situations (Outcome B).

	Model 1B		Model 2B	
**Outcome B**	**β**	**SE**	**β**	**SE**
**Case type**				
Farmer suspects calf pneumonia	ref		ref	
Farmer suspects watery mouth	0.348**	0.110	0.325**	0.108
Farmer wants to prevent calf pneumonia	-0.672***	0.109	-0.698***	0.108
Farmer wants to prevent watery mouth	0.168	0.111	0.151	0.109
**Farmer relationship**				
The farmer is a client of 10 years and the veterinarian rarely visits the farm	ref		ref	
The farmer is a client of 10 years and the veterinarian regularly visits his dairy herd	0.551***	0.096	0.559***	0.096
The farmer has been a client for less than a year	-0.301**	0.094	-0.285**	0.093
**Other veterinarians in practice**				
No other veterinarian in the practice has prescribed the farmer this antibiotic before without consultation	ref		ref	
Other veterinarians in the practice have prescribed the farmer this antibiotic before without consultation	0.572***	0.078	0.574***	0.078
**Time pressure**				
The veterinarian is running late for afternoon consults	ref		ref	
The veterinarian is not running late for afternoon consults	0.355***	0.077	0.355***	0.077
**Habit**				
The farmer uses this antibiotic the same time every year	ref		ref	
The farmer has never used the antibiotic for this reason before	-0.496***	0.077	-0.495***	0.077
**Willingness to pay**				
Farmer does not want to pay for a veterinary visit	ref		ref	
Farmer says he is happy for a veterinary visit	-0.544***	0.077	-0.545***	0.077
**Confidence**				
The veterinarian is confident in the farmers' judgement of the disease	ref		ref	
The veterinarian is not confident in the farmers' judgement of disease	-1.369***	0.079	-1.367***	0.090
**Age**				
< = 30	ref		ref	
>31	-0.438*	0.185	-0.452*	0.184
**Region**				
South East England	ref		ref	
Northern Ireland	3.053***	0.465	3.040***	0.464
Scotland, North West England	1.157***	0.314	1.159***	0.313
Wales, Central, North East, and South West England	0.848**	0.302	0.852**	0.301
**Agreeableness score**				
< = 4	ref		ref	
4.5–5.5	-0.739**	0.233	-0.745**	0.233
> = 6	-0.702**	0.246	-0.706**	0.246
**Intercept**	5.913	0.362	5.935	0.362
**Covariance (slope, intercept)**			-0.234	0.090
**Log likelihood**	-4922.604		-4915.026	
**AIC**	9883.209		9872.052	
	**Marginal**	**Cond.**	**Marginal**	**Cond.**
R^2	0.217	0.504	0.217	0.531
**VPC**	0.366			
**MAE**	1.486		1.411	
**N Vignettes**	2298		2298	

(*Continued*)

Table 4. (Continued)

	Model 1B		Model 2B	
Outcome B	β	SE	β	SE
N Respondents	289		289	

*p≤0.05

**p≤0.01

***p≤0.001. SE = Standard Error; Cond. = Conditional

https://doi.org/10.1371/journal.pone.0213855.t004

prescribe to farmers who have habitual AMU behaviour, such as using antimicrobials for prevention. According to Triandis' Theory of Interpersonal Behaviour, as the frequency of past behaviour increases, the level of consciousness on the decision to perform a behaviour decreases [33]. Thus, prescribing to habitual users of antimicrobials may become an automatic process.

When the farmer was not willing to pay for a farm visit, veterinarians were significantly more likely to prescribe. This is because a situational constraint has been placed on the veterinarian's decision; a construct of the TPB called Perceived Behavioural Control [32]. With the veterinarian unable to visit the farm, they are relying on the farmer's information and cannot carry out a clinical investigation before making the decision. The farmer's decision will affect the veterinarian's perceived ease or difficulty of performing a consultation before prescribing. Similar situational constraints on veterinarians' decisions to prescribe identified from pig practice include farmer management practice and willingness or ability to invest in prevention measures [13].

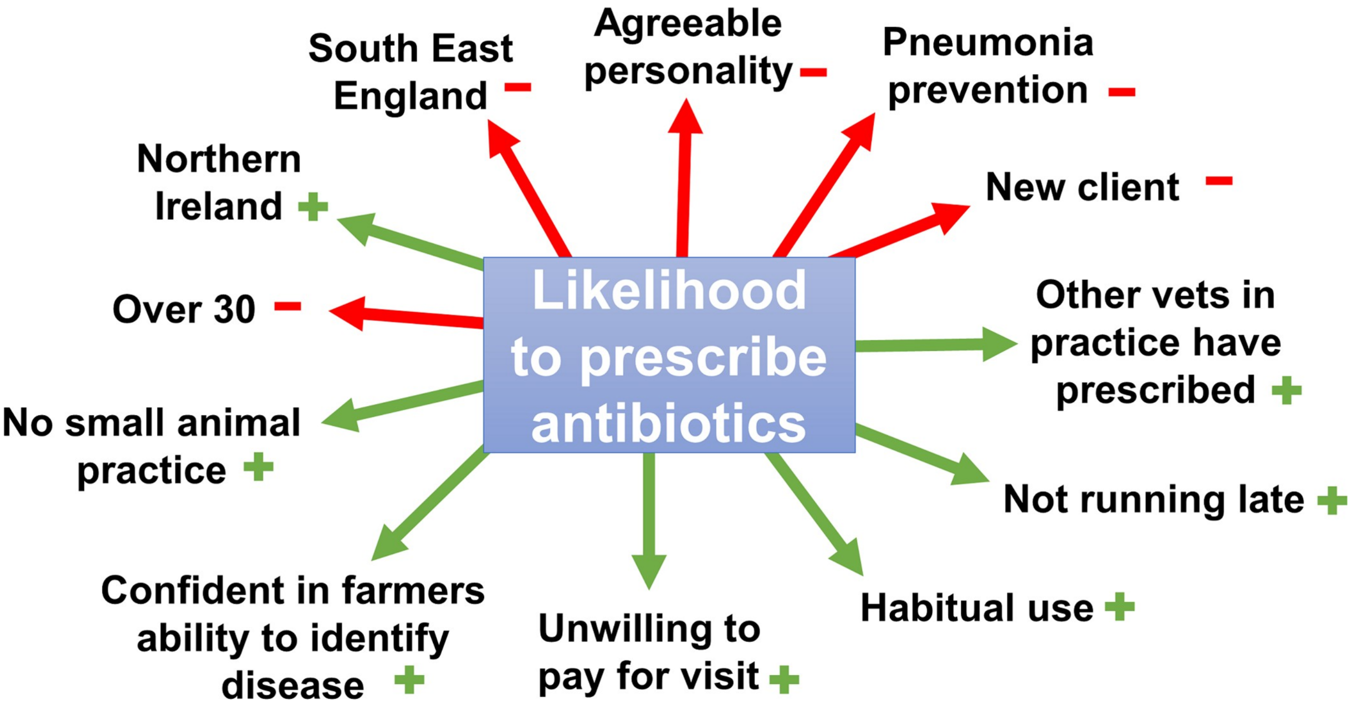

Fig 2. Framework obtained from the results of the survey with regards to significant factors associated with the decision to prescribe antibiotics (+ and— signs indicate direction of associations) (p< = 0.05).

https://doi.org/10.1371/journal.pone.0213855.g002

This study reveals that the prescribing of antimicrobials for the prevention of watery mouth may still be a generally accepted practice by veterinarians, despite UK campaigns communicating that this should not the case [50]. This may be because veterinarians are generally less confident working with sheep than cattle [5, 51], and spend more of their time with cattle (Table 2). Consequently, veterinarians are probably more confident in the alternatives to antimicrobials available to control calf pneumonia, compared with watery mouth in lambs. The practice of prophylactic treatment of watery mouth in lambs is an example of potential avoidable use of antimicrobials, which may select for resistance.

As well as the vignette variables, four veterinarian or veterinary practice characteristics significantly influenced the decision to prescribe. Interestingly, those that rated themselves as more agreeable were significantly less likely to prescribe antimicrobials themselves and expected fewer other veterinarians to do so. Agreeable people are perceived as more benevolent, sociable, and willing to engage in conversations with unfamiliar people which may allow for easier discussions of appropriate alternatives to AMU [52]. Additionally agreeableness increases with age [53], as also indicated in this study ($p \leq 0.001$). With age, veterinarians would be expected to have increased confidence in prescribing behaviour. This may also be why age was significantly associated with the rating for the percentage of other vets who would prescribe, but not with the likelihood to prescribe themselves. Less experienced or younger veterinarians could be more perceptive to norms, or they may think they are more prudent with their prescribing decisions than experienced veterinarians [54], Respondents were significantly less likely to prescribe if their practice had a small animal department. The influence of small animal practice on farm animal practice prescribing behaviour has not been identified before. Antimicrobial use practice policies are no more prominent in small animal practice than in farm animal [55]. It is possible that the variation is simply due the social influence of the different management practices.

Overall, this study identified some areas for improvement in veterinary prescribing behaviour, such as prescribing for the prevention of watery mouth, or prescribing habitually. With new European Union regulations on veterinary medicines coming into place in 2022 [56], veterinarians must be more stringent with their antimicrobial prescribing. For example, prescribing antibiotics for prophylactic treatment will not be allowed except in exceptional circumstances. Additionally, prescribing antimicrobials for routine treatments, or to compensate for poor animal management will not be allowed. Therefore, veterinarians will need to carefully consider how producers will use the antimicrobials they ask for. On the other hand, the study identified areas where veterinary prescribing behaviour was optimal, such as prescribing to farms which have regular visits, prescribing when not under time pressure and the importance of farmer veterinarian relationship in facilitating this. This may highlight that farm animal veterinarians have recently developed a more advisory role on farms, rather than just being present in emergencies. Further research is needed on how trust is built and how veterinarians determine confidence in farmer diagnoses.

Strengths and limitations

This study is novel in its use of a factorial survey approach to measure veterinarian decision-making. The advantage of this method is that it reduces the confounding of social desirability bias by forcing respondents to make trade-offs without direct questioning. This is evident from the results for the variable of case type, where there was no significant difference in the respondent's ratings for cases of suspected calf pneumonia or for the prevention watery mouth in lambs.

The factorial survey approach improves the validity of the study by combining experimental design with survey methods. Applying survey methods means that a broader respondent population can be reached compared to experimental or qualitative research, allowing the results to be more generalisable; therefore increasing external validity. In addition, the experimental design of random allocation of vignettes to respondents increases internal validity.

A limitation of this study is that nearly a quarter of variability in the decision-making was explained by the factors explored, suggesting the possibility of other unexplored factors influencing the decision. This is not uncommon in behaviour research as the most commonly used theory, TPB, tends to explain 19.3% of variability in a behaviour [57]. Various cross-validation and multiple model checks showed that models were robust.

Veterinarians who answered this survey were on average younger than those who responded to the large-scale Survey of the Veterinary Profession [58]. However, many of the demographics, such as gender and role proportions, were similar. Though there were regional prescribing differences, care must be taken when interpreting these results as a small number of responses were received from Northern Ireland, so unlikely to be a representative sample. Nevertheless, this study has one of the largest sample sizes for research into UK veterinarian decision-making regarding antimicrobials to date.

Conclusion

In conclusion, this is the first study to measure the relative importance of factors on the veterinarian's decision to prescribe antimicrobials in contexts other than clinical consultation, as well as the first study to measure veterinarian prescribing decisions to sheep and beef farmers. Social theoretical constructs such as habit, social influence and trust; veterinarian, and veterinary practice characteristics all had an influence on the decision to prescribe. These influential factors could be considered to target interventions in farm animal veterinary practice for improved antimicrobial stewardship. From a 'One Health' perspective, this may help to ensure animal welfare is safeguarded while any impact of veterinary antimicrobial use on antimicrobial resistance in either animal or human disease remains minimal. Further research is needed on how trust is built and how veterinarians determine confidence in farmer diagnoses.

Supporting information

S1 File. Copy of an example survey.
(DOCX)

Acknowledgments

We would like to thank all veterinarians who completed the survey. We thank Dr. Lis King (AHDB) for her useful comments.

Author Contributions

Conceptualization: Charlotte Doidge, Chris Hudson, Fiona Lovatt, Jasmeet Kaler.

Data curation: Charlotte Doidge.

Formal analysis: Charlotte Doidge, Jasmeet Kaler.

Funding acquisition: Jasmeet Kaler.

Investigation: Charlotte Doidge.

Methodology: Charlotte Doidge, Chris Hudson, Fiona Lovatt, Jasmeet Kaler.

Project administration: Charlotte Doidge, Jasmeet Kaler.

Supervision: Chris Hudson, Fiona Lovatt, Jasmeet Kaler.

Validation: Charlotte Doidge.

Visualization: Charlotte Doidge.

Writing – original draft: Charlotte Doidge, Jasmeet Kaler.

Writing – review & editing: Charlotte Doidge, Chris Hudson, Fiona Lovatt, Jasmeet Kaler.

References

1. Tang KL, Caffrey NP, Nóbrega DB, Cork SC, Ronksley PE, Barkema HW, et al. Restricting the use of antibiotics in food-producing animals and its associations with antibiotic resistance in food-producing animals and human beings: a systematic review and meta-analysis. The Lancet Planetary health. 2017; 1(8):e316–e27.
2. Callens B, Cargnel M, Sarrazin S, Dewulf J, Hoet B, Vermeersch K, et al. Associations between a decreased veterinary antimicrobial use and resistance in commensal Escherichia coli from Belgian livestock species (2011–2015). Prev Vet Med. 2018; 157:50–8.
3. O'Neill J. Tackling drug-resistant infections globally: Final report and recommendations. 2016. Available from: https://amr-review.org/sites/default/files/160525_Final%20paper_with%20cover.pdf.
4. Etienne J, Chirico S, Gunabalasingham T, Dautzenberg S, Gysen S. EU Insights–Perceptions on the human health impact of antimicrobial resistance (AMR) and antibiotics use in animals across the EU. EFSA Supporting Publications. 2017; 14(3):1183E.
5. Kaler J, Green LE. Sheep farmer opinions on the current and future role of veterinarians in flock health management on sheep farms: A qualitative study. Prev Vet Med. 2013; 112(3):370–7.
6. Lowe P. Unlocking Potential: A Report on Veterinary Expertise in Food Animal Production: to the Vets and Veterinary Services Steering Group: Vets and Veterinary Services Steering Group; 2009.
7. Lima E, Hopkins T, Gurney E, Shortall O, Lovatt F, Davies P, et al. Drivers for precision livestock technology adoption: A study of factors associated with adoption of electronic identification technology by commercial sheep farmers in England and Wales. PLOS ONE. 2018; 13(1):e0190489. https://doi.org/10.1371/journal.pone.0190489 PMID: 29293617
8. Veterinary Medicines Directorate. UK Veterinary Antibiotic Resistance and Sales Surveillance Report 2017. 2018.
9. Davies P, Remnant JG, Green MJ, Gascoigne E, Gibbon N, Hyde R, et al. Quantitative analysis of antibiotic usage in British sheep flocks. Vet Rec. 2017; 181(19):511. https://doi.org/10.1136/vr.104501 PMID: 29051311
10. Coyne L, Pinchbeck G, Williams N, Smith R, Dawson S, Pearson R, et al. Understanding antimicrobial use and prescribing behaviours by pig veterinary surgeons and farmers: a qualitative study. Vet Rec. 2014; 175(23):593. https://doi.org/10.1136/vr.102686 PMID: 25200432
11. Mateus ALP, Brodbelt DC, Barber N, Stärk KDC. Qualitative study of factors associated with antimicrobial usage in seven small animal veterinary practices in the UK. Prev Vet Med. 2014; 117(1):68–78. https://doi.org/10.1016/j.prevetmed.2014.05.007 PMID: 25091861
12. Speksnijder D, Jaarsma A, Gugten A, Verheij TJ, Wagenaar J. Determinants associated with veterinary antimicrobial prescribing in farm animals in the Netherlands: a qualitative study. Zoonoses Public Health. 2015; 62(s1):39–51.
13. Coyne LA, Latham SM, Williams NJ, Dawson S, Donald IJ, Pearson RB, et al. Understanding the culture of antimicrobial prescribing in agriculture: a qualitative study of UK pig veterinary surgeons. J Antimicrob Chemother. 2016; 71(11):3300–12. https://doi.org/10.1093/jac/dkw300 PMID: 27516473
14. Visschers VH, Backhans A, Collineau L, Loesken S, Nielsen E, Postma M, et al. A comparison of pig farmers' and veterinarians' perceptions and intentions to reduce antimicrobial usage in six European countries. Zoonoses Public Health. 2016; 63(7):534–44. https://doi.org/10.1111/zph.12260 PMID: 26890125
15. Coyne LA, Latham SM, Dawson S, Donald IJ, Pearson RB, Smith RF, et al. Antimicrobial use practices, attitudes and responsibilities in UK farm animal veterinary surgeons. Prev Vet Med. 2018; 161:115–26. https://doi.org/10.1016/j.prevetmed.2018.10.021 PMID: 30466652
16. De Briyne N, Atkinson J, Pokludová L, Borriello SP, Price S. Factors influencing antibiotic prescribing habits and use of sensitivity testing amongst veterinarians in Europe. Vet Rec. 2013; 173(19):475–. https://doi.org/10.1136/vr.101454 PMID: 24068699

17. Busani L, Graziani C, Binkin N, Franco A, Di Egidio A, Battisti A. Survey of the knowledge, attitudes and practice of Italian beef and dairy cattle veterinarians concerning the use of antibiotics. Vet Rec. 2004; 155(23):733–8. PMID: 15623086

18. Gibbons J, Boland F, Buckley J, Butler F, Egan J, Fanning S, et al. Influences on antimicrobial prescribing behaviour of veterinary practitioners in cattle practice in Ireland. Vet Rec. 2013; 172(1):14. https://doi.org/10.1136/vr.100782 PMID: 23293148

19. Walker AE, Grimshaw JM, Armstrong EM. Salient beliefs and intentions to prescribe antibiotics for patients with a sore throat. Br J Health Psychol. 2001; 6(4):347–60.

20. Eccles MP, Grimshaw JM, Johnston M, Steen N, Pitts NB, Thomas R, et al. Applying psychological theories to evidence-based clinical practice: Identifying factors predictive of managing upper respiratory tract infections without antibiotics. Implement Sci. 2007; 2:26. https://doi.org/10.1186/1748-5908-2-26 PMID: 17683558

21. Godin G, Bélanger-Gravel A, Eccles M, Grimshaw J. Healthcare professionals' intentions and behaviours: A systematic review of studies based on social cognitive theories. Implement Sci. 2008; 3(1):36.

22. Hagger MS, Chatzisarantis NLD. An Integrated Behavior Change Model for Physical Activity. Exerc Sport Sci Rev. 2014; 42(2):62–9. https://doi.org/10.1249/JES.0000000000000008 PMID: 24508739

23. Killick C, Taylor BJ. Judgements of Social Care Professionals on Elder Abuse Referrals: A Factorial Survey. Bri J Soc Work. 2012; 42(5):814–32.

24. Lyons CJ. Individual perceptions and the social construction of hate crimes: A factorial survey. Soc Sci J. 2008; 45(1):107–31.

25. Chatfield SL, Nolan R, Crawford H, Hallam JS. Acute care nurses' responses and recommendations for improvement of hand hygiene compliance: A cross-sectional factorial survey research study. Am J Infect Control. 2017; 45(6):620–5. https://doi.org/10.1016/j.ajic.2016.12.024 PMID: 28233570

26. Taylor BJ. Factorial Surveys: Using Vignettes to Study Professional Judgement. Bri J Soc Work. 2006; 36(7):1187–207.

27. Kuhfeld WF. Discrete choice. SAS Technical Papers. 2010; 2010:285–663.

28. Auspurg K, Hinz T. Factorial survey experiments. London: Sage Publications; 2014.

29. Pardon B, De Bleecker K, Hostens M, Callens J, Dewulf J, Deprez P. Longitudinal study on morbidity and mortality in white veal calves in Belgium. BMC Vet Res. 2012; 8(1):26.

30. RUMA. RUMA Targets Task Force Report. 2017.

31. Morgan RM, Hunt SD. The Commitment-Trust Theory of Relationship Marketing. J Mark. 1994; 58 (3):20–38.

32. Ajzen I. The theory of planned behavior. Organ Behav Hum Decis Process. 1991; 50(2):179–211.

33. Triandis HC. Interpersonal behavior: Brooks/Cole Pub. Co.; 1977.

34. Cialdini RB, Kallgren CA, Reno RR. A focus theory of normative conduct: A theoretical refinement and reevaluation of the role of norms in human behavior. Adv Exp Soc Psychol. 1991; 24:201–34.

35. Busemeyer JR, Townsend JT. Decision field theory: a dynamic-cognitive approach to decision making in an uncertain environment. Psychol Rev. 1993; 100(3):432. PMID: 8356185

36. Gosling SD, Rentfrow PJ, Swann WB. A very brief measure of the Big-Five personality domains. J Res Pers. 2003; 37(6):504–28.

37. Faul F, Erdfelder E, Buchner A, Lang A-G. Statistical power analyses using G* Power 3.1: Tests for correlation and regression analyses. Behav Res Methods. 2009; 41(4):1149–60. https://doi.org/10.3758/BRM.41.4.1149 PMID: 19897823

38. Charlton C, Rabash J, Browne W, Healy M, Cameron B. MLwiN. 3.02 ed. University Of Bristol: Centre for Multilevel Modelling; 2017.

39. Nakagawa S, Schielzeth H. A general and simple method for obtaining R2 from generalized linear mixed-effects models. Methods Ecol Evol. 2013; 4(2):133–42.

40. Johnson PC. Extension of Nakagawa & Schielzeth's R2GLMM to random slopes models. Methods Ecol Evol. 2014; 5(9):944–6. https://doi.org/10.1111/2041-210X.12225 PMID: 25810896

41. Barton K. MuMIn: Multi-Model Inference. 2018.

42. R Core Team. R: A language and environment for statistical computing. Vienna, Austria2018.

43. Jansen J, Steuten CDM, Renes RJ, Aarts N, Lam TJGM. Debunking the myth of the hard-to-reach farmer: Effective communication on udder health. J Dairy Sci. 2010; 93(3):1296–306. https://doi.org/10.3168/jds.2009-2794 PMID: 20172249

44. Higgins HM, Golding SE, Mouncey J, Nanjiani I, Cook AJC. Understanding veterinarians' prescribing decisions on antibiotic dry cow therapy. J Dairy Sci. 2017; 100(4):2909–16. https://doi.org/10.3168/jds.2016-11923 PMID: 28131572

45. Ruston A, Shortall O, Green M, Brennan M, Wapenaar W, Kaler J. Challenges facing the farm animal veterinary profession in England: A qualitative study of veterinarians' perceptions and responses. Prev Vet Med. 2016; 127:84–93. https://doi.org/10.1016/j.prevetmed.2016.03.008 PMID: 27094145
46. EMA & EFSA. EMA and EFSA Joint Scientific Opinion on measures to reduce the need to use antimicrobial agents in animal husbandry in the European Union, and the resulting impacts on food safety (RONAFA). 2016.
47. Björkman I, Erntell M, Röing M, Lundborg CS. Infectious disease management in primary care: perceptions of GPs. BMC Fam Prac. 2011; 12(1):1.
48. Lucas PJ, Cabral C, Hay AD, Horwood J. A systematic review of parent and clinician views and perceptions that influence prescribing decisions in relation to acute childhood infections in primary care. Scand J Prim Health Care. 2015; 33(1):11–20. https://doi.org/10.3109/02813432.2015.1001942 PMID: 25716427
49. Lewis PJ, Tully MP. Uncomfortable prescribing decisions in hospitals: the impact of teamwork. J R Soc Med. 2009; 102(11):481–8. https://doi.org/10.1258/jrsm.2009.090150 PMID: 19875537
50. RUMA. Targets Task Force Report 20172017. Available from: http://www.ruma.org.uk/wp-content/uploads/2017/10/RUMA-Targets-Task-Force-Report-2017-FINAL.pdf.
51. Bellet C, Woodnutt J, Green LE, Kaler J. Preventative services offered by veterinarians on sheep farms in England and Wales: Opinions and drivers for proactive flock health planning. Prev Vet Med. 2015; 122(4):381–8. https://doi.org/10.1016/j.prevetmed.2015.07.008 PMID: 26255688
52. Roccas S, Sagiv L, Schwartz SH, Knafo A. The Big Five Personality Factors and Personal Values. Pers Soc Psychol Rev. 2002; 28(6):789–801.
53. Roberts BW, Wood D, Smith JL. Evaluating five factor theory and social investment perspectives on personality trait development. J Res Pers. 2005; 39(1):166–84.
54. Hopman NEM, Hulscher MEJL, Graveland H, Speksnijder DC, Wagenaar JA, Broens EM. Factors influencing antimicrobial prescribing by Dutch companion animal veterinarians: A qualitative study. Prev Vet Med. 2018; 158:106–13. https://doi.org/10.1016/j.prevetmed.2018.07.013 PMID: 30220383
55. Hughes LA, Williams N, Clegg P, Callaby R, Nuttall T, Coyne K, et al. Cross-sectional survey of antimicrobial prescribing patterns in UK small animal veterinary practice. Prev Vet Med. 2012; 104(3):309–16.
56. Regulation (EU) 2019/6 of the European Parliament and of the Council of 11 December 2018 on veterinary medicinal products and repealing Directive 2001/82/EC, 107 (2019).
57. McEachan RRC, Conner M, Taylor NJ, Lawton RJ. Prospective prediction of health-related behaviours with the Theory of Planned Behaviour: a meta-analysis. Health Psychology Review. 2011; 5(2):97–144.
58. Buzzeo J, Robinson D, Williams M. The 2014 RCVS Survey of the Veterinary Profession 2014. Available from: https://www.employment-studies.co.uk/resource/2014-rcvs-survey-veterinary-profession.
